# Digital economy and urban economic resilience: The mediating role of technological innovation and entrepreneurial vitality

**DOI:** 10.1371/journal.pone.0303782

**Published:** 2024-06-28

**Authors:** Suping Xiao, Ping Zhou, Lin Zhou, Sike Wong

**Affiliations:** 1 Department of Business Administration, Zhejiang Institute of Administration, Hangzhou, China; 2 School of Business, Guangdong University of Foreign Studies, Guangzhou, China; 3 School of Business, Guangdong Polytechnic Normal University, Guangzhou, China; 4 Shenzhen Polytechnic University, Shenzhen, China; Babes-Bolyai University, Cluj-Napoca, ROMANIA

## Abstract

Drawing on the diffusion of innovation theory, we argue that the development of digital economy has a positive effect on urban economic resilience. Using panel data from 284 cities in China from 2011 to 2018, we empirically examine the relationship between digital economy and urban economic resilience. We find a positive and significant link between them, mediated by technological innovation and entrepreneurial vitality. Moreover, the heterogeneity analysis shows that the impact of digital economy is most pronounced in smaller cities, with its effects diminishing in larger cities and megacities. Our results underscore the importance and the direction of fostering digital economy development.

## 1 Introduction

Economic resilience represents the ability of regions to resist external shocks, adapt to changes, and recover from shocks [1], and is the key to regional competitive advantage. In recent years, the global economy has been struggling to cope with unexpected disruptions, including the economic contraction caused by COVID-19, market turmoil caused by local conflicts and trade frictions. The escalating frequency of such unpredictable adversities is challenging the resilience of regional economies. In response to these risks and shocks, some regions struggle to recover, while others demonstrate the ability to withstand external shocks, swiftly recover, and even achieve resource reallocation and industrial transformation by adapting to these shocks. This stark contrast in risk response capabilities has piqued scholarly interest, making urban economic resilience a burgeoning research topic.

Existing studies on economic resilience mainly analyze the antecedents of economic resilience from the perspective of internal characteristics and external conditions of the economic system. Studies from the internal perspective have explored the impact of economic agglomeration, industrial structure, and industrial relevance on economic resilience. According to Zhang et al. (2021) [2], industry diversification agglomeration enhances urban economic resilience during shock resistance periods. However, during recovery and adjustment periods, industry specialization agglomeration improves urban economic resilience, while diversified agglomeration does not have a positive impact. Novak et al. (2021) explored the impact of three key dimensions of supply chain resilience (preparedness, alertness, and flexibility) on firm financial performance and found that preparedness had a greater impact on financial performance than alertness and flexibility [3]. Rocchetta and Mina (2019) found that regions with consistent and not merely diversified knowledge bases exhibit greater adaptive resilience to unexpected economic downturns [4].

Studies from the external perspective have analyzed the impact of environmental conditions on economic resilience, such as social capital, regional innovation ability, and so on. Östh et al. (2018) find that the presence of social capital and transport accessibility significantly influences the resilience of urban areas in terms of economic development, education, age, ethnic composition, and poverty [5]. Bristow and Healy (2018) indicate that regions identified as innovation leaders during the 2007–2008 crisis were more likely to resist or recover quickly [6]. Kong et al. (2022) review the relationship between urban resilience and various functions, like social capital and resource accessibility, and highlight the importance of innovation capacity in facilitating rapid recovery from economic shocks [7]. However, prior research on economic resilience has not fully considered the interaction and synergy effects between internal and external factors caused by digital technology changes, ignoring the complexity of economic systems.

**Fig 1:**
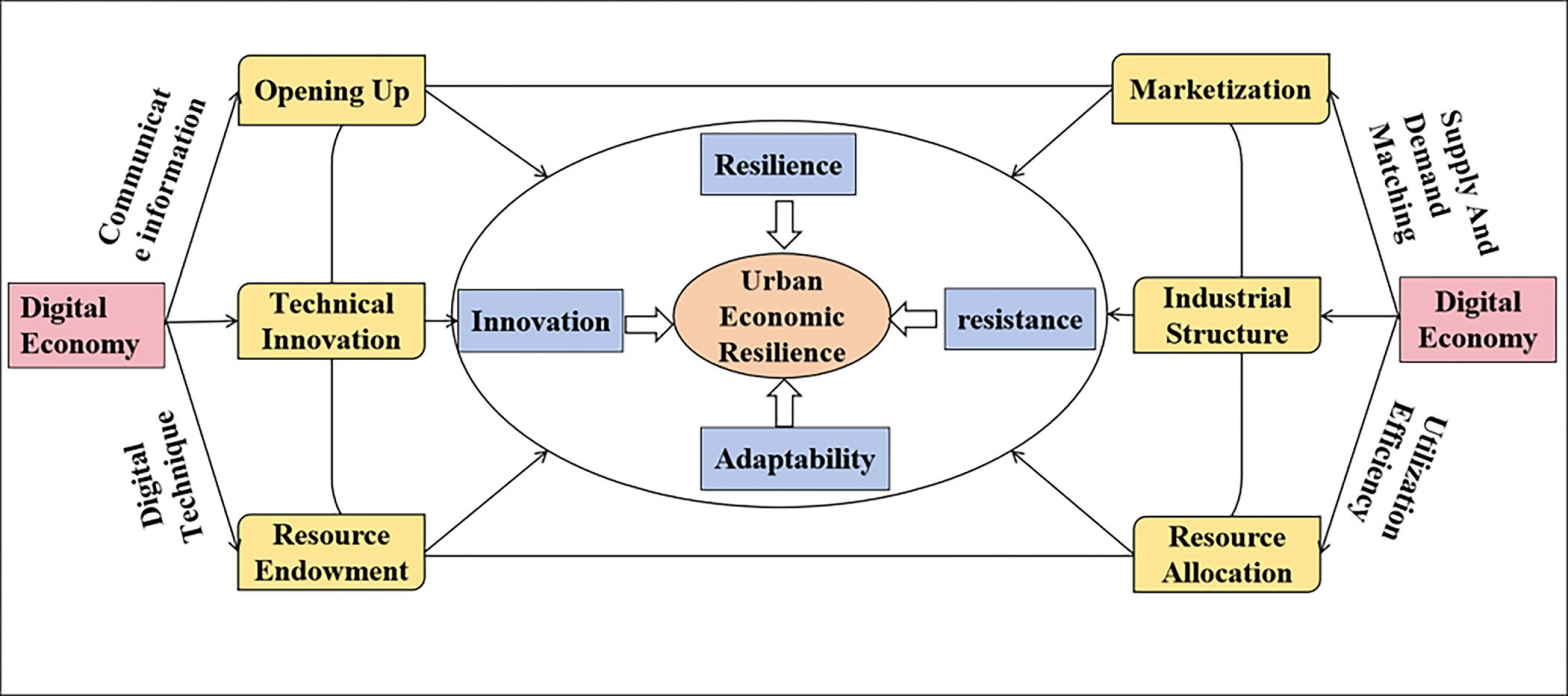
The impact of the digital economy on urban economic resilience.

The digital economy, driven by the widespread application of digital technologies such as big data, the Internet of Things, and cloud computing, has gradually become the “new engine” of economic growth. These emerging digital technologies have transformed the pattern of urban economic development by reducing costs, improving efficiency, and enhancing the flexibility and adaptability of market entities. Many researchers have agreed that the digital economy promotes economic growth and improves economic efficiency. For instance, Zhang et al. (2021) found that the development of the digital economy can effectively improve the supply and demand structure, enhance production efficiency, and thus drive the endogenous growth of the economy [8]. Kumar et al. (2023) and Zhang et al. (2023) indicated that digital advancements have enhanced the efficiency of urban economic activities by facilitating real-time communication and the flexibility and adaptability of market entities, leading to faster innovation cycles and more responsive market mechanisms [9,10]. To clarify the factors influencing economic resilience, we depicted Fig 1.

However, the impact of the digital economy on economic resilience, which refers to the ability of an economy to withstand or recover from various shocks and crises, is relatively unexplored. Only a few studies have examined the relationship between the digital economy and economic resilience [11,12], and they have not fully explored the underlying mechanisms and the moderating effects of city characteristics. Therefore, this paper aims to address the following research questions: (1) How does the digital economy affect urban economic resilience? (2) What are the main mechanisms through which the digital economy influences urban economic resilience? (3) How do city characteristics, such as size and region, moderate the relationship between the digital economy and urban economic resilience? These questions are of great significance for updating our understanding of urban economic resilience in the context of digital transformation, which is a profound event that most economies are experiencing.

To answer these questions, we examine the impact of the digital economy on urban economic resilience based on the theory of diffusion of innovation. First, we established a digital economy indicator system using panel data from 284 Chinese cities from 2011 to 2018. Then, we investigate the impact of digital economy development on the economic resilience of cities and examine the mediating role of technological innovation and entrepreneurial vigor. Finally, we explored the heterogeneous impact of the digital economy on urban economic resilience from the perspective of city size. Our contribution is mainly reflected in (1) supplementing the research on the antecedents of economic resilience; (2) expanding the research on the digital economy by investigating the mechanism underlying the relationship between the digital economy and economic resilience; and (3) providing empirical evidence for constructing urban economic resilience.

The rest of the paper is organized as follows: the second part mainly reviews the relevant literature and proposes the research hypotheses; the third part is the data source and research design, which introduces the measurement and model design; the fourth part is the empirical results and analysis; the fifth part is the research conclusion and discussion.

## 2 Literature review and hypothesis development

The digital economy, as a new economic form after the agricultural economy and industrial economy, is a new driving force for urban economic development [2,13] and a new path for cities to realize breakthrough evolution. On the one hand, the digital industry spawned by the development of digital technology has enhanced the level of intelligence and modularity of city evolution on the basis of promoting the integration of factors and enhancing the efficiency of factor utilization [2,14,15]. On the other hand, the diffusion of digital technology innovations has also promoted the transformation and upgrading of traditional industries, fundamentally changing the technological and socio-cultural environment of the urban economy [16].

### 2.1 The direct impact of the digital economy on urban economic resilience

Diffusion of innovation theory is a theory used to explain the process and law of the diffusion and adoption of new ideas, new things, and new technologies in the social system [15]. Diffusion of innovations theory believes that the characteristics of the innovations themselves and the channels of diffusion affect the speed and scope of diffusion of innovations. Innovative technologies with obvious relative advantages and strong compatibility can be applied and popularized faster [17].

In the theoretical lens of diffusion of innovation theory, digital technology is characterized by obvious advantages and strong compatibility. Saleem et al.(2023) revealed that AI adoption strengthens adaptive resilience and moderates the role of IoT edge in German family-owned SMEs [18]. Ghobakhloo (2020) identifies factors influencing the implementation of Information and Digital Technologies (IDT) in smart manufacturing and highlights the importance of digital technology and management support [19]. Skare et al.(2023) used the Digital Economy and Society Index (DESI) to explore how digital transformation impacts SMEs and found SMEs benefit from the collective pool of digital knowledge [20]. The interplay between these technologies not only enhances operational efficiency and productivity but also paves the way for new business models, services, and revenue streams, thereby reshaping traditional industries and fostering economic diversification [21]. Moreover, digital technology has been emphasized and supported by the government. For example, the Chinese central government founded a big data bureau to coordinate data resources and the application of data technology in various fields in 2023.

The diffusion of digital technology can bring about the integration and innovation of elements, and promote the diversity of the urban economic system and the synergy between different elements. The deep integration of digital technology with the real economy has given rise to new industries, new business forms, and new models, promoting the diversification of the industrial structure. The diversified industrial structure can play the role of a "shock absorber" in the face of external shocks, which effectively reduces risk concentration and shares external shocks among industries [2,22], thus increasing the resilience of the urban economic system.

The diffusion of digital technologies also promotes the dissemination and sharing of information across time and space, increasing the informatization and intelligence of urban economic systems. This interconnection and information sharing can increase the degree of association of existing production factors [20], and improve the perception and early warning capabilities of urban economic systems. Thereby various entities within a city can respond to risks more quickly when experiencing external shocks, enhancing the stability of economic systems on the whole. For instance, when a torrential rainstorm disaster struck Henan province, China on July 20, 2021, a university student posted a shared document that matched requests for help with information about relief resources and advanced disaster relief in a timely and effective manner, demonstrating the important role of digital technologies in enhancing resilience. Thus, we propose:

**Hypothesis 1:** Digital economy development is positively associated with urban economic resilience.

### 2.2 The indirect impact of the digital economy on urban economic resilience

#### The mediating role of technological innovation

The digital economy can promote technological innovation. The digital economy itself is characterized by technological innovation and technological accumulation [4]. The digital economy has empowered market entities, from small businesses to large corporations, to be more agile and adaptable to the ever-changing economic landscape, thus bolstering their resilience to volatility [21]. Moreover, the digital economy can improve the efficiency of resource matching by reducing the cost of innovation search and realizing more effective knowledge production, which in turn improves the overall level of innovation in society [23,24].

The present study examines how technological innovation contributes to economic resilience from two perspectives. Firstly, technological innovation stimulates the emergence of new processes, technologies, and regional innovation systems by enabling the inter-regional and inter-industrial transfer of innovation factors and knowledge. Moreover, the development of digital technology can significantly improve the industrial structure in the region [25]. This results in the reconfiguration of resources and the creation of new economic growth trajectories [26,27]. Secondly, technological innovation enhances the market performance and environment by endowing products with competitive advantages in terms of cost and quality, which augment the risk-bearing and shock-absorbing capacities of market actors. As a result, we propose

**Hypothesis 2:** The digital economy indirectly affects urban economic resilience by promoting technological innovation.

#### The mediating role of entrepreneurial vitality

On the demand side, digital technology enables a variety of remote services and collaboration, which enlarges the entrepreneurial space [28]. Furthermore, digital technology lowers the cost of product or service customization, which boosts the demand for personalized offerings [29]. Petersen et al. (2022) demonstrated that the personalization and trade growth induced by the digital economy broadens the range of entrepreneurial opportunities [30].

On the supply side, digital technologies facilitate the emergence of sharing economy platforms and the improvement of digital infrastructures, which refine the division of labor [31,32]. This reduces the operational cost for micro and small enterprises and thus lowers the entry barrier for entrepreneurship. Sahut et al. (2021) indicated that the development of the digital economy promotes entrepreneurship and increases the success rate of entrepreneurial ventures by enhancing the efficiency of collaborative R&D, production, sales, and distribution [33].

With the diffusion of digital infrastructure, any individual can access domestic and international markets via the Internet and benefit from the advances in information technology [34]. More and more entrepreneurs are selling niche products or services through platforms such as TikTok or Temu, which leads to flourishing entrepreneurial activities. Entrepreneurship not only helps entrepreneurs and self-employed individuals raise their income but also positively affects social equality and economic resilience [35]. Thus, we propose

**Hypothesis 3:** Digital economy development indirectly influences urban economic resilience by fostering entrepreneurial vitality.

**Fig 2:**
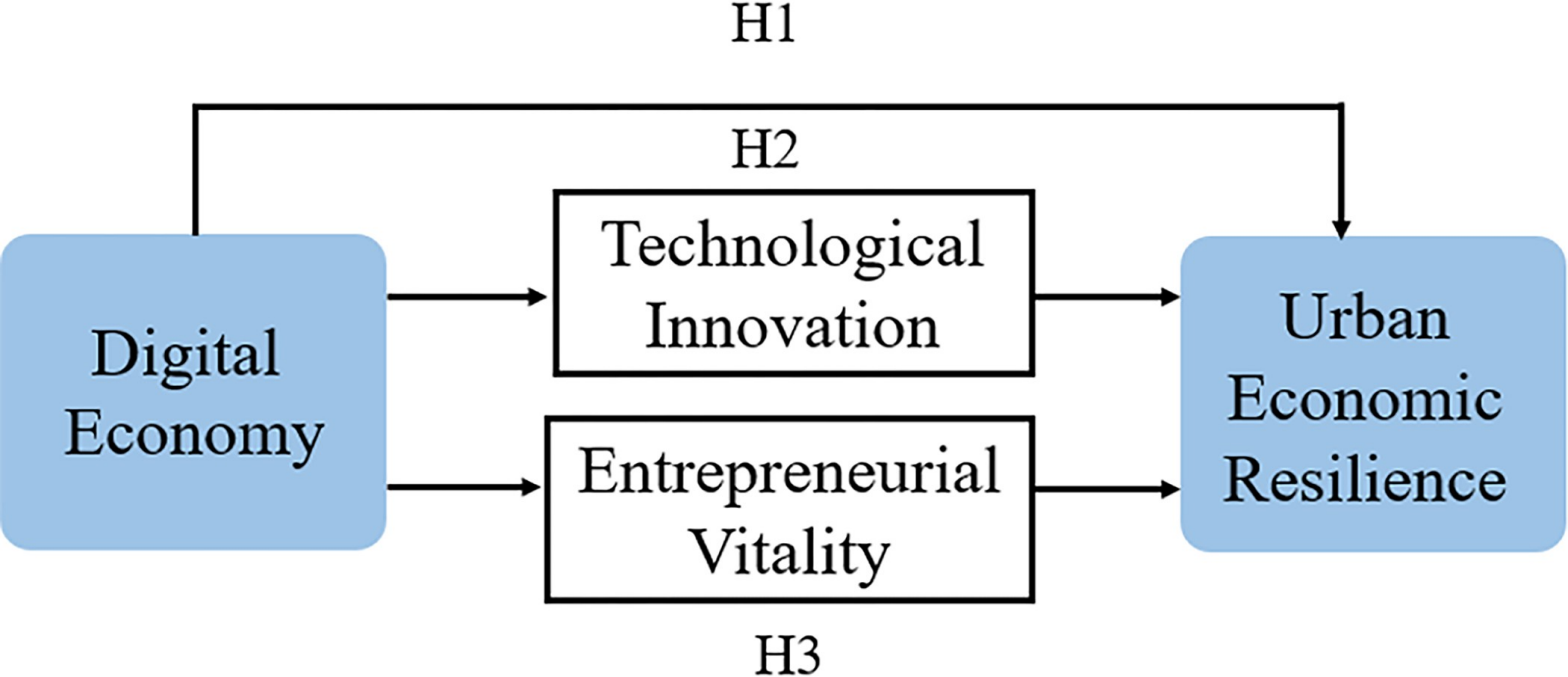
Theoretical model.

To illustrate our hypothesis, we depicted Fig 2.

## 3 Methodology

### 3.1 Samples and data sources

As China has undergone rapid digital development in the past decade and can be a rich context for our research question, we choose panel data consisting of 284 cities in China from 2011–2018. The sample consists of panel data from 284 cities in China from 2011–2018. The data on the digital finance index are from the Peking University Digital Inclusive Finance Index (PKU-DFIIC) (2011–2018). The data on e-commerce are from the "China Taobao Village Research Report (2011–2018)" published by Ali Research Institute. The data of surviving enterprises in digital-related industries are obtained from the WIND database. Patent data are from the China Patent Database of the State Intellectual Property Office. Other data are from China City Statistical Yearbook, China Regional Economic Statistical Yearbook, and China Environment Statistical Yearbook. Some missing data were supplemented by manually collected from provincial and municipal statistical yearbooks and statistical bulletins.

Relevant data underlying our findings is in the supporting information files.

### 3.2 Variables and measurement

1. Dependent variable

Urban economic resilience (*UER*). In prior studies, employment rate and change in GDP are the two most common methods to measure urban economic resilience [11]. In this study, we measure the level of urban economic resilience by the employment rate. Specifically, we use the logarithm of the proportion of the number of those employed in public and private sectors and self-employed workers to the total population at the end of the year as the measure of urban economic resilience.

2. Independent variable

Digital economy development (*DE*). Following Ma and Zhu (2022) [14], we computed the index of digital economy development using the entropy method. The measurements are as follows

Step one: the data are normalized. For positive indicators, the measure is $x_{ij}^{\prime }=(x_{ij}-\bar{x_{j}})/s_{j}$; for negative indicators, the measure is $x_{ij}^{\prime }=(\bar{x_{j}}{-x}_{ij})/s_{j}$. *x*_*ij*_ is the original value of the ith sample, *j* indicator, $x_{ij}^{\prime }$ represents the standardized value, $\bar{x}$ and *s*_*j*_ are the mean and standard deviation of the *j* indicator, respectively. Due to the need for logarithmic processing in the entropy value method, some of the values after standardization cannot be used directly as they are negative. Thus, we pan the standardized value: $z_{ij}=x_{ij}^{\prime }+A$, where A takes the value of 0.0001.

Step two: each indicator is processed dimensionless, and then the entropy value method is used to assign weights to the indicators. First, we calculated the value of the *j*th indicator of the *i*th city as a proportion of the overall indicator *p*_*ij*_: $p_{ij}=z_{ij}/\sum_{i=1}^{n}z_{ij}$ (i = 1,2,···,n; j = 1,2,···,m), where *n* is the number of cities and *m* is the number of indicators. Second, we calculated the entropy value of the *j*th indicator: $\alpha_{j}=-k\sum_{i=1}^{n}z_{ij}ln(p_{ij}),\;k=1/ln(n)$, *α*_*j*_≥0 and calculate its coefficient of variance *ε*_*j*_ = 1−*α*_*j*_. Further, we normalized the coefficient of variance to derive the weight of the *j*th indicator: $w_{j}=\varepsilon_{j}/\sum_{j=1}^{m}\varepsilon_{j}$ (j = 1,2,···,m). Lastly, the digital economy development level of each city is finally derived: $F_{i}=\sum_{j=1}^{m}w_{j}p_{ij}$。

The indicator system for the level of digital economy development includes the dimensions shown in Table 1.

**Table 1:** Evaluation of digital economy development.

	Primary Indicators	Secondary indicators	Measurement
Digital economy	Infrastructure, production factor index	Internet penetration rate	Number of Internet broadband subscribers per 100 people
		Cell phone penetration rate	Number of cell phone subscribers per 100 people
		Human Capital	Number of college students per 10,000 people
		IT industry employment	Number of employees in the IT industries
	Industry development index	E-commerce	"Taobao village" number
		Information transmission, software, and information services industries	Number of surviving companies in the software and IT services, Internet, and related services
		Telecommunications industry	Total telecom business per capita
		Digital finance	Peking University Digital Inclusive Finance Index

3. Mediating variables

Technological innovation. Following Wu et al. (2023) [36], the logarithm of the number of patents per 10,000 people was used to measure technological innovation.

Entrepreneurial vitality. Following Ye et al. (2018) [37], we logarithmically processed the ratio of the number of newly created and currently surviving private enterprises to the total number of enterprises at the end of the year to measure entrepreneurial vitality in each city.

4. Control variables

Based on the existing literature, we controlled for the following variables that may affect urban economic resilience: (1) the industrial structure (*Ind*). Cainelli et al. (2019) found a significant effect of the industrial structure on regional economic resilience by analyzing the factors influencing the economic resilience of the European Union (EU) region during the financial crisis of 2008–2012 [38]. Thus, we controlled for *Ind*, which is measured by the logarithm of the proportion of the third industry output in regional GDP; (2) the degree of openness to the outside world (*FDI*). Canh & Thanh (2020) showed that the degree of trade openness has a significant impact on the economic resilience of the region [39]. We measured *FDI* by the logarithm of the ratio of the actual amount of foreign capital (converting to RMB using the exchange rate of previous years) to regional GDP; (3) government intervention (*Gov*). Mavrodieva et al. (2019) analyzed the role of government actions on economic resilience and found that government subsidies to SMEs can effectively enhance the level of economic recovery in cities [40]. We measured government intervention by the logarithm of the ratio of total expenditure in the local fiscal budget to GDP; (4) urban infrastructure (*Infra*). A study by Allam and Jones (2019) with a sample of small-scale cities found that cities with higher levels of infrastructure development were able to achieve economic recovery more quickly after a disaster [41]. Thus, we controlled urban infrastructure by the logarithm of urban paved road area per capita; (5) urbanization (*Urban*). Wang et al. (2022) found a significant positive correlation between urbanization and economic resilience [42]. We measured urbanization by the logarithm of the ratio of the number of urban populations to the total population of each city; (6) marketization (*mark*et). We matched the provincial-level marketization index by Wang et al. (2017) [43] with the corresponding cities.

### 3.3 Fixed-effect MODELS models

To test the impact of the digital economy on urban economic resilience, we designed the following model.


$${UER}_{it}=\alpha +\alpha_{1}{DE}_{it}+\alpha_{2}{control}_{it}+\mu_{i}+\lambda_{t}+\varepsilon_{it}$$ (1)

In Eq (1), *i* denotes the city, *t* denotes the year, and *UER*_*it*_ denotes the city i’s economic resilience at *t*, and *DE*_*it*_ denotes city i’s digital economy development at time *t*. *control*_*it*_ is a series of control variables. *μ*_*i*_、*λ*_*t*_ represent the regional fixed effects and time fixed effects, respectively, and *ε*_*it*_ is the random error term.

To test the mediating effect of entrepreneurial vitality and technological innovation, we followed the lead of Baron and Kenny (1986) [44] and used a stepwise regression method. The models are specified in Eqs (2) and (3).


$${Mediate}_{it}=\beta +\beta_{1}{DE}_{it}+\beta_{2}{control}_{it}+\mu_{i}+\lambda_{t}+\varepsilon_{it}$$ (2)

$${UER}_{it}=\gamma +\gamma_{1}{DE}_{it}+{\gamma_{2}Mediate}_{it}+\gamma_{3}{control}_{it}+\mu_{i}+\lambda_{t}+\varepsilon_{it}$$ (3)

In Eqs (2) and (3), *Mediate*_*it*_ represents technological innovation and entrepreneurial vitality respectively. Eq (2) examines the effect of digital economy development on the mediators, and Eq (3) examines whether technological innovation and entrepreneurial vitality play a mediating role in the relationship between digital economy development and urban economic resilience. Control variables are the same as in Eq (1).

## 4 Results

### 4.1 Descriptive statistics

As shown in Table 2, the descriptive analysis shows that the mean value and SD of urban economic resilience are -1.171 and 1.827 respectively, suggesting that the sample cities vary significantly in terms of economic resilience.

**Table 2:** Descriptive statistics.

Variables	Sign	MEAN	SD	Min	Max
Urban economic resilience	*UER*	-1.171	1.827	-6.108	8.671
Digital economy development	*DE*	-8.159	0.756	-10.029	-4.224
Industry structure	*Ind*	-0.942	0.244	-2.288	-0.211
Openness to the outside	*FDI*	-4.140	2.071	-13.553	2.985
Government intervention	*Gov*	-2.600	0.351	-4.787	-1.269
Urban infrastructure development	*Infra*	1.199	0.897	-3.386	4.291
Marketization	*Market*	1.914	0.295	-2.813	2.491
Urbanization	*Urban*	-0.656	0.273	-2.763	0
Technology innovation	*Tec*	1.269	1.357	-3.570	5.652
Entrepreneurial vitality	*Entre*	-2.104	0.332	-5.278	-0.079

### 4.2 Baseline regression

To control for potential heteroskedasticity as well as serial correlation, we used a fixed-effect model for regression analysis, and the standard errors of the regression coefficients are clustered and adjusted at both time and region levels to mitigate the serial correlation problem. The regression results of the fixed-effect model are shown in Table 3. Model (1) shows that the regression coefficient of *DE* is significantly positive (*β* = 0.751, *t* = 11.77), and R^2^ is 0.2078 without control variables. Model (2) shows that after entering control variables, the coefficient of *DE*, although decreasing, is still significantly positive (*β* = 0.616, *t* = 8.02), and R^2^ is improved to 0.2821, suggesting that digital economy development enhances urban economic resilience. Hypothesis 1 is supported.

**Table 3:** The impact of the digital economy on urban economic resilience.

	(1)	(2)
	*UER*	*UER*
*DE*	0.751*** (11.77)	0.616*** (8.02)
*Ind*		-0.575*** (-2.95)
*FDI*		-0.204*** (-8.21)
*Gov*		0.111* (0.94)
*Infra*		0.396*** (6.70)
*Market*		0.583*** (4.26)
*Urban*		-0.150* (-0.76)
*Region*	Yes	Yes
*Year*	Yes	Yes
Constant	4.707*** (8.49)	2.829*** (4.08)
R^2^	0.2078	0.2821
N	2272	2272

Note: *, **, and*** indicate statistical significance at 10%, 5%, and 1%, respectively.

T-values are reported in parentheses.

As to the regression results of the control variables, there is a significant negative correlation between industrial structure and urban economic resilience, which is consistent with the current research [38]. When an economy suffers from external shocks, the negative impact is more likely to spread to related industries, generating a chain reaction that negatively influences economic resilience. The coefficient of urbanization on urban economic resilience is significantly negative. Urbanization is generally believed to be conducive to promoting economic agglomeration, which normally has a positive effect on economic resilience. Lu et al. (2023) argued that urbanization can increase economic resilience because urbanization promotes economic growth, innovation, diversification, and efficiency, as well as increases human, social, and institutional capital, which improves the ability of cities to cope with economic crises, as evidenced by their empirical study using China’s county abolition reform practices from 2007–2019 and a multi-temporal DID model [45]. Yet, due to China’s rapid and rough urbanization pattern, the "big city" disease may be prominent. Urbanization can lead to overconsumption of resources, environmental degradation, rising inequalities, and difficulties in governance, thereby increasing the risks and challenges faced by cities. As a result, urbanization may reduce urban economic resilience as shown in our empirical results.

Government intervention, the level of urban infrastructure development, and marketization all have a significant positive relationship with urban economic resilience. Government investment is not only beneficial to economic growth but also indicates financial assistance to cities in the face of external shocks and thus enables economic recovery. The level of urban infrastructure development and marketization can help lower the barriers to factor mobility, increase the rate of mobility, and optimize the allocation of factor resources in the region, thus enhancing the economic resilience of cities.

### 4.3 The mediation effect

We predicted that digital economy development may affect urban economic resilience by driving technological innovation and enhancing entrepreneurial vitality in Hypotheses 2 and 3.

Regarding the mediating effect of technological innovation, as shown in Table 5 Column (1), the coefficient of *DE* on technological innovation is significantly positive (*β* = 0.463), indicating that the digital economy development boosts technological innovation in cities.

Compared to the baseline regression, Table 4 Column (2) shows that the coefficient of *DE* is 0.538 at the 1% significant level, smaller than the coefficient of 0.611 as reported in the baseline regression in Table 3, suggesting that digital economy development enhances urban economic resilience through technological innovation. Hypothesis 2 is supported.

**Table 4:** The mediating effects of technological innovation and entrepreneurial vitality.

	Dependent variables			
	*Tec*	*UER*	*Entre*	*UER*
	(1)	(2)	(3)	(4)
*DE*	0.463*** (11.48)	0.538** (6.36)	0.027** (1.34)	0.565*** (7.70)
*Tec*		0.099** (2.06)		
*Entre*				0.081** (1.61)
*Ind*	-0.113(-1.30)	-0.542*** (-2.83)	0.041 (0.89)	-0.578*** (-2.97)
*FDI*	0.008 (0.98)	-0.212*** (-8.64)	0.001 (0.10)	-0.204*** (-8.24)
*Gov*	0.088* (1.72)	0.109 (0.94)	0.021** (0.75)	0.110 (0.93)
*Infra*	0.528*** (18.24)	0.351*** (5.78)	-0.020 (-1.39)	0.398*** (6.74)
*Market*	0.633*** (8.23)	-0.711*** (-4.84)	-0.026(-0.73)	-0.577*** (-4.22)
*Urban*	0.584*** (6.54)	-0.209 (-1.05)	-0.144*** (-3.07)	-0.099(-0.50)
*Region*	Yes	Yes	Yes	Yes
*Year*	Yes	Yes	Yes	Yes
*Constant*	3.507*** (8.88)	2.314*** (3.22)	-1.938*** (-10.56)	3.153*** (4.39)
R^2^	0.6921	0.2839	0.1931	0.2828
N	2272	2272	2272	2272

Note: *, **, and*** indicate statistical significance at 10%, 5%, and 1%, respectively.

T-values are reported in parentheses.

In terms of entrepreneurial vitality, Table 4 Column (3) shows that the coefficient of *DE* is significantly positive (*β* = 0.027), implying that digital economy development significantly enhances urban entrepreneurial vitality. After entering entrepreneurial vitality in Column (4), the regression on urban economic resilience shows that the coefficient of *DE* is positive (*β* = 0.565) at 1% significant level, indicating that entrepreneurial vitality plays a partial mediating effect in the process of digital economy development affecting urban economic resilience. As such, Hypothesis 3 is supported. The digital economy affects urban economic resilience by promoting technological innovation and stimulating entrepreneurial vitality.

To further examine the robustness of the mediating effects, we conducted the Sobel test and bootstrap analysis. The results are shown in Table 5. Sobel test results indicate that the effect of the digital economy on urban economic resilience is significantly affected by the level of technological innovation (Z = 2.011, *P* <0.05) and entrepreneurial vitality (Z = 1.25, *P* <0.05). Moreover, the total value of mediating effects reveals that both mediating paths of technological innovation and entrepreneurial vitality are partially mediated, so the digital economy may affect urban economic resilience through other mechanisms. In addition, the bootstrap estimation results in Table 5 show that the 95% confidence intervals of the two paths are [0.063, 0.153] and [0.018, 0.058], respectively, which demonstrates the significance of the mediating effects of technological innovation and entrepreneurial vitality.

**Table 5:** Sobel test and Bootstrapped indirect effects.

		Technology Innovation	Entrepreneurial Vitality
Sobel test	Sobel value	0.070** (Z = 2.011)	0.018** (Z = 1.25)
	Total Intermediary Effect	0.843	0.593
Bootstrap	95% Confidence Interval	[0.063, 0.153]	[0. 018, 0.058]
	Intermediary Effect Value	0.071** (1.65)	0.038*** (3.74)

Note: *, **, and*** indicate statistical significance at 10%, 5%, and 1%, respectively. Bootstrapped sample size = 5000.

### 4.4 Robustness checks

The following robust analysis has been added to test the robustness of the results. (1) We use an alternative measure of digital economy development. Referring to the China Internet+ Report (2015–2018), we use the China Internet+ composite index as the measure of the digital economy development level and rerun our model. The estimation results, presented in Table 6, show that the coefficient of the digital economy is significantly positive (*β* = 0.167, *t* = 1.74), demonstrating that digital economy development has a consistent effect on urban economic resilience.

**Table 6:** Robust tests: Digital economy development and urban economic resilience.

	(1) Remeasurement of *DE*	(2) Two-stage IV regression
*DE*	0.167**(1.74)	0.479*** (4.78)
*Ind*	0.155*(1.81)	-0.953***(-2.88)
*FDI*	0.008(0.21)	-0.162***(-6.81)
*Gov*	0.025**(2.04)	0.189(1.12)
*Infra*	0.673***(9.71)	0.528***(5.33)
*Market*	-0.097***(-3.58)	-1.068 ***(-4.40)
*Urban*	0.005(1.08)	-0.338(-1.04)
*Constant*	-1.157***(-2.90)	3.047***(3.19)
R^2^	0.2261	0.1646
Region	Yes	
Year	Yes	
Kleibergen—Paaprk LM		323.764 [0.0000]
Cragg—Donald Wald F		Significance level<10%
Hansen J		10.274 [0.130]
N	1136	1704

Note: *, **, and*** indicate statistical significance at 10%, 5%, and 1%, respectively.

T-values are reported in parentheses.

(2) We use instrumental variable regression to test the robustness of our findings. Although our research has controlled for many variables that may affect urban economic resilience, such as industrial structure, FDI, and urbanization level, there may be other missing factors that influence urban economic resilience. To mitigate endogeneity concerns arising from missing variables, we use two-stage IV regression with the interaction of the number of post offices (fixed telephones) in Chinese cities in 1984 and the amount of completed fixed asset investment in the Internet information services sector in each city as instrumental variables. The logic is as follows. First, the level of digital economy development is closely related to infrastructure such as post offices and fixed telephones, satisfying the correlation requirement of the instrumental variable. Second, the number of post offices and fixed telephones in a city does not have a direct impact on urban economic resilience. Thus, the number of post offices and telephones may be a valid instrument variable. The number of post offices and the number of fixed telephones in cities is available only in 1984. Following Nunn and Qian’s (2014) [46], we multiply it by the amount of completed fixed asset investment in the Internet information services sector in each city, a variable that reflects time change, to construct a panel of instrumental variables.

IV Step One:

$${DE}_{it}=\alpha \delta +\delta_{1}{post}_{it}+\delta_{2}{telep}_{it}+\delta_{3}{control}_{it}+\mu_{i}+\lambda_{t}+\varepsilon_{it}$$ (4)

IV Step Two:

$${UER}_{it}=\rho +\rho_{1}{DE}_{it}+\rho_{2}{control}_{it}+\mu_{i}+\lambda_{t}+\varepsilon_{it}$$ (5)

Table 6 presents the regression results of two-stage IV modeling. The first-stage regression shows that the number of post offices and the number of telephones in the city in 1984 significantly and positively related to digital economy development in the city. Moreover, the Cragg-Donald Wald F-statistic (20.797) is much larger than 10, which excludes the problem of "weak instrumental variables". The Kleibergen-Paaprk LM test passed the 1% significance level and the Hansen J value was insignificant, so the problems of "non-identification" and "over-identification" of the selected instrumental variables were excluded, which also indicated that both instrumental variables satisfied the exogenous requirement. The second-stage regression results show that the impact of digital economy development on economic resilience is significantly positive, consistent with the baseline regression results, thus further validating our main findings.

### 4.5 Heterogeneity analysis

To further analyze the heterogeneity impact of the digital economy on urban economic resilience among cities of different sizes, we classified our sample cities into four categories: small cities, medium cities, large cities, and mega-cities according to the criteria of *Notice on Adjusting the Criteria for Classifying City Size* issued by the State Council in 2014. Then we used fixed effects estimation methods to examine the heterogeneous effects of the digital economy on urban economic resilience.

As shown in Table 7 Columns (5) to (8), heterogeneity analysis indicates that digital economy development has a significant positive impact on urban economic resilience in all other cities except medium-sized cities, with the greatest impact in small cities and the impact decreasing in large, mega-cities. The possible reason for the difference is that smaller cities tend to have a relatively weak foundation and a low starting point in developing the digital economy. The development of the digital economy may mitigate the weakness of small cities in restrained local markets and small populations and play a more important role in boosting urban economic resilience. In contrast, the digital economy development in large cities has limited space to further enhance urban economic resilience, where the level of industrial structure diversification and technological innovation is already high.

**Table 7:** Heterogeneity analysis.

Variables	Small Cities	Medium-sized cities	Large Cities	Megacities
	(1)	(2)	(3)	(4)
*DE*	2.754*** (2.71)	0.143 (0.15)	0.874*** (7.38)	0.462*** (3.99)
*Ind*	0.670 (0.41)	5.149*** (2.90)	-0.572** (-2.56)	-0.769** (-2.05)
*FDI*	-0.136 (-1.49)	-0.449** (-2.03)	-0.149*** (-4.67)	-0.078* (-1.80)
*Gov*	-1.432 (-1.30)	-3.097** (-2.21)	0.074 (0.54)	0.179 (1.06)
*Infra*	-0.150 (-0.17)	1.205* (1.58)	0.056 (0.75)	0.228** (2.50)
*Market*	-6.309** (-2.58)	-2.558** (-2.09)	-0.303** (-2.05)	-0.354 (-1.30)
*Urban*	-0.105 (-0.06)	-2.066 (-1.35)	0.244 (1.09)	0.453** (1.27)
Region	Yes	Yes	Yes	Yes
Time	Yes	Yes	Yes	Yes
*Constant*	25.154** (2.27)	-3.357 (-0.40)	5.410*** (5.24)	2.226** (2.49)
R^2^	0.9421	0.5998	0.2511	0.2602
N	34	71	1374	793

Note: *, **, and*** indicate statistical significance at 10%, 5%, and 1%, respectively.

T-values are reported in parentheses.

## 5 Discussion

Based on panel data from 284 cities in China from 2011 to 2018, we empirically examined the impact of digital economy development on urban economic resilience and the mediating role of technological innovation and entrepreneurial vitality using fixed effects models and hierarchical regression methods. Our findings are as follows. (1) The development of the digital economy is positively associated with urban economic resilience, and the conclusion holds after controlling for endogeneity problems. (2) The underlying mechanisms through which the development of the digital economy enhances urban economic resilience are technological innovation and entrepreneurial vitality. (3) The heterogeneity analysis shows that the digital economy has the most pronounced effect on urban economic resilience for small cities, a weaker effect for large cities and megacities, and an insignificant effect for medium-sized cities. By examining these mediating variables, our study provides a more comprehensive and nuanced understanding of how digital economy development affects urban economic resilience.

### 5.1 Theoretical contributions

First, our study supplements the research on the antecedents of economic resilience. Previous studies have explored the factors that affect economic resilience from the internal characteristics and external conditions of the economic system independently. Yet, they fail to account for how technological change affects the interplay and mutual reinforcement of factors within and outside the economy, largely overlooking the complexity of economic systems. Our study fills this gap by examining how digital economy development influences economic resilience at the city level in China, a country that has experienced rapid digital transformation and urbanization in the past decade. We also conduct heterogeneity analysis to examine the different effects of the digital economy on urban economic resilience across different city sizes, which offers a more nuanced understanding of the effect of digital economy development on urban economic resilience.

Second, our study extends research on digital economy by investigating its impact on urban economic resilience. Prior research on the digital economy has predominantly concentrated on its influence on key economic factors such as economic growth, productivity, and employment. For instance, Zeshan et al. (2021) explored the role of an enabling HRM digital system in mediating this relationship between digitalization and employees’ autonomy, addressing the autonomy paradox associated with information technology [47]. Other scholars have explored the implications of the digital economy on social dimensions, including the potential for increased inequality and its impact on the environment [48,49]. These studies have provided valuable insights into the multifaceted effects of digitalization on both the economy and society at large. However, there is a noted gap in the literature regarding the relationship between the digital economy and urban economic resilience. Our study, by examining whether and how the digital economy affects urban economic resilience, complements the literature on the consequences of digital economy from a new perspective.

Thirdly, our study complements research on the macro consequences of digital economy development by exploring the mediating roles of technological innovation and entrepreneurial vitality in the relationship between digital economy development and urban economic resilience. Saleem et al. (2023) explored how AI adoption, LoT edge, and adaptive resilience interact to foster digital innovation, but they did not consider the role of entrepreneurship as a potential outcome of digital economy development [50,51]. Therefore, our study fills this gap by proposing and testing a theoretical model that links digital economy development with urban economic resilience through technological innovation and entrepreneurial vitality.

### 5.2 Managerial implications

The results lead to several policy implications. First, our study reveals that digital economy development enhances urban economic resilience. Therefore, policymakers could encourage the diffusion and adoption of digital technologies in various sectors of the urban economy, such as e-commerce, e-government, e-health, etc., to boost urban economic resilience.

Second, our study suggests that driving technological innovation and stimulating entrepreneurial vitality may be important mechanisms through which the digital economy affects urban economic resilience. Thus, government departments should take measures to encourage people to explore application scenarios of digital technology and support the development of technological innovation and entrepreneurial vitality in cities by providing financial incentives, infrastructure facilities, human capital development, intellectual property protection, etc., to stimulate the creation and diffusion of new ideas, products, and business models.

Third, policymakers should pay attention to the different needs and challenges of cities of different sizes in terms of digital economy development. For instance, small cities could provide more tailored and targeted policies and programs, such as fostering a digital collaboration ecosystem by establishing innovation hubs, to enhance digital capabilities and competitiveness.

## 6 Conclusion

By analyzing panel data from 284 cities in China from 2011 to 2018, we have demonstrated that digital economy development enhances urban economic resilience by promoting technological innovation and entrepreneurial vitality. We hope that this study will advance the understanding of the relationship between digital economy and its macro consequences.

Our study has some limitations. First, due to data availability limitations, we did not include indicators reflecting public concern about digital privacy and data security in our measurement of digital economy development. However, these indicators may have impacts on technology innovation and entrepreneurial vitality, and economic resilience. Therefore, we suggest that future research could use more refined indicators of digital economy to capture its various aspects and impacts. Second, our analysis is based on panel data of 284 cities in China from 2011 to 2018. However, this period may not be long enough to capture the long-term effects of digital economy development on urban economic resilience. Future research could extend the span of the data and examine the dynamics in the relationship between digital economy development and urban economic resilience.

## Supporting information

S1 Data (XLSX)
